# Transcriptome‐Based Analysis of Core Hub Genes Related to Cell Adhesion in Vero Cells

**DOI:** 10.1155/bmri/1265156

**Published:** 2026-05-23

**Authors:** Yiyang Fan, Yan Wang, Ruixiang Bi, Yapeng Zhao, Wanning Gao, Derong Zhang, Qiongyi Li, Jialin Bai

**Affiliations:** ^1^ Engineering Research Center for Key Technology and Industrialization of Cell-Based Vaccine, Ministry of Education, Northwest Minzu University, Lanzhou, China, xbmu.edu.cn; ^2^ Key Laboratory of Bioengineering and Biotechnology of the National Ethnic Affairs Commission, Biomedical Research Center, Northwest Minzu University, Lanzhou, China, xbmu.edu.cn; ^3^ Gansu Technological Innovation Center of Animal Cell, Biomedical Research Center, Northwest Minzu University, Lanzhou, China, xbmu.edu.cn; ^4^ College of Life Science and Engineering, Northwest Minzu University, Lanzhou, China, xbmu.edu.cn

**Keywords:** genetic engineering technique, hub gene, PPI, suspension cell lines, Vero cells

## Abstract

The key to constructing suspension cell lines through genetic engineering technology lies in identifying the fundamental components and molecules involved in cell adhesion. In this study, differentially expressed genes between adherent and suspension Vero cells were analyzed using transcriptomics. The biological processes and pathways associated with these differentially expressed genes were examined through GSEA, GO, and KEGG enrichment analyses. The STRING database was employed to construct a protein–protein interaction (PPI) network, and core hub genes were identified using the MCODE and CytoHubba plugins in Cytoscape. Six core hub genes related to cell adhesion in Vero cells were identified: *ITGA3*, *ITGA5*, *ITGB3*, *ITGB5*, *JUP*, and *PECAM1*.

## 1. Introduction

Animal cell culture refers to the technology that enables the continuous growth, proliferation, and differentiation of cells in vitro within an artificially simulated in vivo environment [1]. Based on the culture method, it can be classified into adherent culture and suspension culture. The establishment of in vitro animal cell culture technology is marked by Harrison′s development of the cover‐covered concave glass hanging drop culture method [2], which was subsequently followed by the introduction of the flask culture method, single‐cell isolation culture, and monolayer cell culture techniques. Among these, monolayer cell culture remains one of the most widely used methods today [3]. The successful mass production of the polio vaccine through cell culture significantly advanced large‐scale culture technology. Consequently, various large‐scale and industrial culture techniques have been developed, including hybridoma technology, microcarrier culture, suspension culture, genetic engineering, serum‐free culture, and three‐dimensional (3D) cell culture technology [4].

Vero cells, a continuous cell line derived from the kidneys of normal adult African green monkeys, are among the best options due to their relative ease of culture, high susceptibility to a variety of viruses, and reputation as a stable means of viral production [5]. Consequently, Vero cells are widely regarded as the preferred cell line for human vaccine production [6]. Since 1986, the World Health Organization (WHO) has approved the use of low‐passage Vero cells for producing various viral vaccines for human use [7]. This approval is based on the fact that Vero cells are heteroploid and capable of multiple divisions, maintain normal cellular functions, and have potential tumorigenicity [8]. Improving productivity while reducing costs remains a primary concern in the biopharmaceutical industry [9]. To address these challenges, suspension culture has been introduced and is gaining popularity among researchers; this method allows for higher cell densities and is cost‐effective [10]. Suspension technology in animal cell culture originated in the early 1950s [11]. Pioneering research by Owens et al. successfully employed a “rolling tube” device to suspend and grow mouse fibroblasts [12]. Earle and Jay cultured 929‐L cells in modified roller tubes rotating on different rollers [13]. However, the first adaptation of Vero cells to suspension growth was reported by Litwin in 1992 [14]. He demonstrated that suspension cultures of Vero cell aggregates could serve as an alternative for large‐scale vaccine production. This work laid the foundation for further development of cell lines for biological production.

To meet the global demand for vaccines, suspension culture technology of Vero cells has remained a prominent research focus [15]. Currently, suspension adaptation and gene editing technologies are the primary methods used to develop suspension cell lines [16]. Suspension adaptation of Vero cells is mainly achieved by gradually reducing serum concentration in the medium and adding growth factors to decrease cell adhesion [17]. Additionally, microcarrier technology can be employed to facilitate cell adaptation to suspension growth, ultimately enabling scale‐up culture in bioreactors. Sunyan et al. cultured Vero cells in square flasks using VP‐SFM and DMEM supplemented with 5% bovine serum to produce the rabies virus CTN‐1 V strain. They found that the rabies virus titer was independent of cell passage number and serum concentration in the medium [18]. Using the Cytodex microcarrier bioreactor, Rourou et al. successfully increased Vero cell density to 5.5 × 10^6^ cells/mL, achieving a maximum rabies virus titer of 3.5 × 10^7^ FFU/mL [19]. In recent years, with advances in biotechnology and genetic engineering, gene editing has emerged as a novel approach to generate suspension cell lines [20]. Cloutier et al. demonstrated that the genes *Siat7e* and *Lama4* are involved in cell adhesion and that modifying their expression affects the adhesion properties of HeLa cells [21].

The key to constructing suspension cell lines using gene editing technology lies in identifying genes that are closely associated with cell adhesion. By modulating the expression of these genes—either upregulating or downregulating them—the adhesion properties of cells can be altered. Therefore, this study focused on analyzing the differential gene expression between adherent and suspension Vero cells through transcriptomic analysis. We performed Gene Set Enrichment Analysis (GSEA), Gene Ontology (GO), and Kyoto Encyclopedia of Genes and Genomes (KEGG) pathway enrichment analyses. Additionally, the STRING database was utilized to investigate protein–protein interactions (PPIs) and to construct a protein interaction network. Core hub genes were identified through further analysis. This work provides a foundation for the development of Vero cell suspension lines.

## 2. Materials and Methods

### 2.1. Cell Lines and Cell Culture

Vero cells were obtained from the American Type Culture Collection (ATCC, Cat. No. CCL‐81, RRID: CVCL_0059). These cells were cultured in DMEM supplemented with 10% newborn bovine serum (Lanzhou Minhai Bioengineering Company) and 1% antibiotics (Lanzhou Minhai Bioengineering Company) at 37°C in a 5% CO_2_ atmosphere. The Vero suspension cell line was developed by Ling Shixin, a senior engineer at the Biomedical Research Center of Northwest Minzu University, using the traditional method of gradually reducing serum concentration. These cells were cultured in serum‐free Vero 601 suspension medium (Shenzhen Yishengke) supplemented with 2% fetal bovine serum (Lanzhou Minhai Bioengineering Company) at 37°C, 5% CO_2_, and 110 rpm on a cell shaker [22].

### 2.2. Data Collection

In the NCBI database (https://www.ncbi.nlm.nih.gov/gene), 593 related genes were obtained with the green monkey as the species and the adhesion gene as the keyword.

### 2.3. RNA‐Seq

Total RNA from each group of cells was extracted using RNAiso Plus reagent (Takara, Dalian, China). Adherent cultured Vero cells were labeled as Adh Vero 1, Adh Vero 2, and Adh Vero 3, whereas suspension‐cultured Vero cells were labeled as Sus Vero 1, Sus Vero 2, and Sus Vero 3. RNA concentration was measured using a trace nucleic acid and protein detector. Qualified total RNA samples were sent to Shanghai Ouyi Biomedical Technology Co. Ltd. for sequencing. DNA was digested with deoxyribonuclease (DNase) to remove contaminating DNA, and mRNA was enriched and fragmented into short segments using magnetic beads with Oligo(dT). First‐strand cDNA was synthesized and purified using a kit to obtain double‐stranded cDNA. After PCR amplification, quality was assessed using an Agilent 2100 Bioanalyzer. Libraries were sequenced on an Illumina HiSeq 2500 sequencer. Raw data were processed and trimmed using Trimmomatic software to ensure accuracy. Data analysis was performed using HTSeq‐count and Cufflinks software. Gene expression levels of protein‐coding genes were calculated using the FPKM (fragments per kilobase of transcript per million mapped reads) method.

### 2.4. Identification of Differentially Expressed Genes (DEGs)

DEGs were analyzed using the DESeq2 package (Version 1.44.0) in RStudio (Version 4.4.1). The *p* values were adjusted to control for false positives. DEGs were identified using a threshold of *p* < 0.05 and |logFC| ≥ 1. The distribution of these genes was visualized using a volcano plot generated with the ggplot2 package (Version 3.5.1). Genes present both in the NCBI database and among the DEGs in Vero cells were considered adhesion‐related DEGs.

### 2.5. Functional Analysis of DEGs

Functional annotation using GO and the KEGG was performed with the Database for Annotation, Visualization, and Integrated Discovery (DAVID). These tools offer functional interpretations for large gene lists derived from genomic studies, with a significance threshold set at *p* < 0.05.

### 2.6. GSEA

To gain a deeper understanding of the biological processes (BPs) involved in cell adhesion, we performed GSEA using the clusterProfiler package in R. Iconic and canonical pathway gene sets were obtained from the Molecular Signatures Database (MSigDB), and an adjusted *p* value of less than 0.05 was used as the cutoff criterion.

### 2.7. PPI Network Construction and Module Analysis

The STRING database was utilized to investigate the relationships among the proteins of interest and to construct a PPI network. Interactions with a combined score greater than 0.4 were considered statistically significant [23]. Cytoscape software (Version 3.9.1) was employed to visualize the protein interaction network. The built‐in Molecular Complex Detection (MCODE) algorithm was used for module analysis, and the six algorithms available in the CytoHubba plug‐in were applied to identify the core genes.

### 2.8. Quantitative Real‐Time (qRT)‐PCR

Total RNA was extracted using RNAiso Plus reagent. cDNA was synthesized using the Evo M‐MLV Reverse Transcription Kit (AG Bio, Hunan, China), and qRT‐PCR was performed using the Applied Biosystems 7500 system (Thermo Fisher Scientific, Inc.). The thermal cycling conditions were as follows: predenaturation at 94°C for 30 s, followed by 40 cycles of denaturation at 94°C for 5 s and annealing at 60°C for 30 s. *GAPDH* was used as the reference gene. Gene‐specific qRT‐PCR primers are listed in Table 1.

**Table 1:** Primers used for the qRT‐PCR experiment.

Gene ID	Primer sequence (5 ^′^–3 ^′^)
PECAM1	Forward: ACTCAGTCATGGCCATGGTG
	Reverse: CGTTGACGATGATGCTGCTG
ITGA3	Forward: TGGTGACATCAACCAGGATGG
	Reverse: ACAGAGATGGGAATCTGCTGGG
ITGA5	Forward: TTACGGGACTCAACTGCACC
	Reverse: CTCCGGGCATTTCAGGATCT
JUP	Forward: GATCGGGAGCAGTAGCCACA
	Reverse: ATGATGCCCTTGCTGCTGAC
ITGB5	Forward: GCTCGCACTCCTGGCTATCTG
	Reverse: CTGGATCGCTCGGCTCTGGAAC
ITGB3	Forward: CGTCACTGCAACAATGGCAA
	Reverse: TTGACCACAGAGACATCGC
GAPDH	Forward: GAGTACGTCGTGGAGTCCACTG
	Reverse: CAAACATAGGGGCGTCAGCAGAG

### 2.9. Statistical Analysis

All analyses in this article were conducted using RStudio, the SangerBox platform, DAVID, and GraphPad Prism 9.5. Data visualization was performed using the Wei Shengxin platform. Relative gene expression levels were calculated using the 2^−*Δ* *Δ*Ct^ method.

## 3. Results

### 3.1. Identification of DEGs

**Figure 1:**
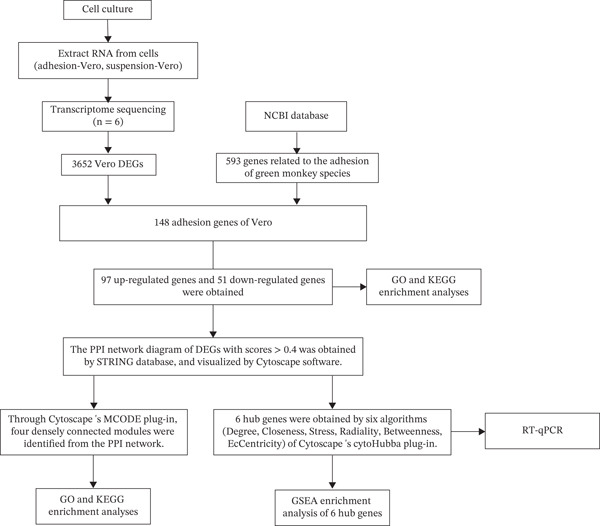
Experimental research flow chart.

The overview of the research flow is shown in Figure 1. A total of 20,093 genes were identified from the two cell lines. Differential expression analysis revealed 3652 DEGs, including 2122 upregulated and 1530 downregulated genes. The volcano plot and heat map of DEGs in Vero cells were generated using the ggplot2 and heat map packages in RStudio (Figure 2a,b). Venn diagram analysis identified 148 overlapping CA‐DEGs between the Vero cell DEGs and the NCBI database (Figure 2c). The expression heat maps of these 148 CA‐DEGs effectively distinguish adherent Vero cells from suspended Vero cells (Figure 2d).

Figure 2Differential gene expression level distribution map. (a) Volcano plots of the identified DEGs from Adh Vero and Sus Vero. Red dots represent upregulated genes screened based on logFC > 1 and *p* < 0.05; blue dots represent downregulated genes screened based on logFC < −1 and *p* < 0.05; grey dots indicate genes with no significant difference. (b) Cluster heat map of differentially expressed genes. (c) Venn diagram of the identified DEGs from Vero cells and *Chlorocebus sabaeus* adhesion genes. (d) Cluster heat map of 148 CA‐DEGs.

**Figure:**
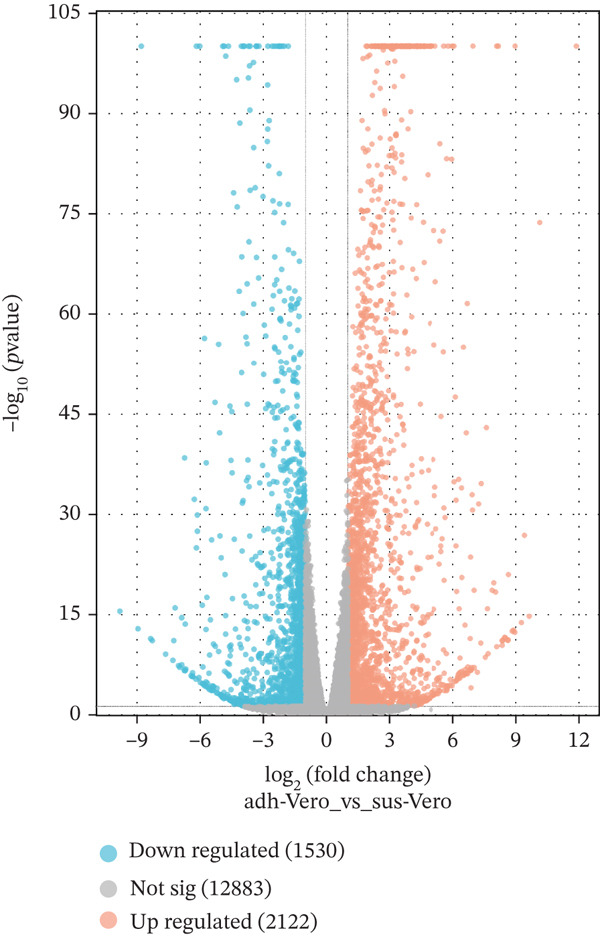
(a)

**Figure:**
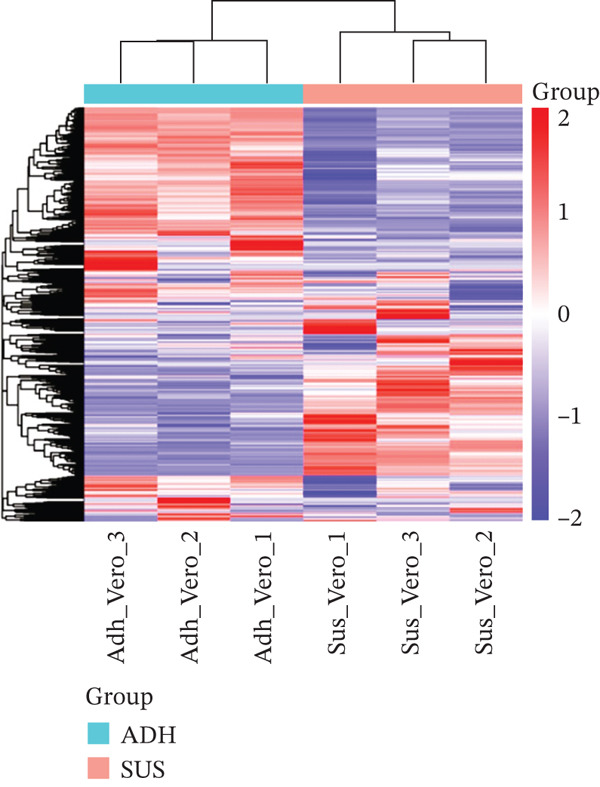
(b)

**Figure:**
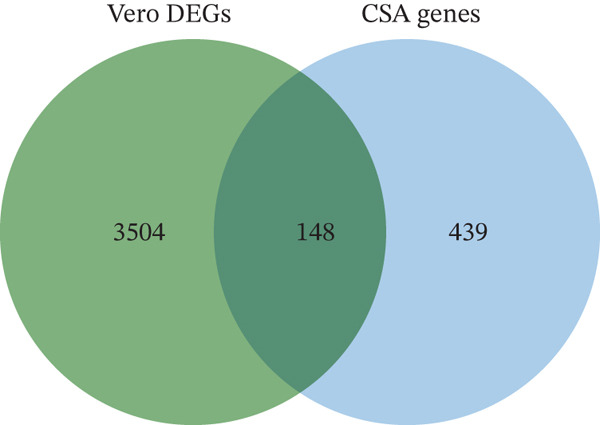
(c)

**Figure:**
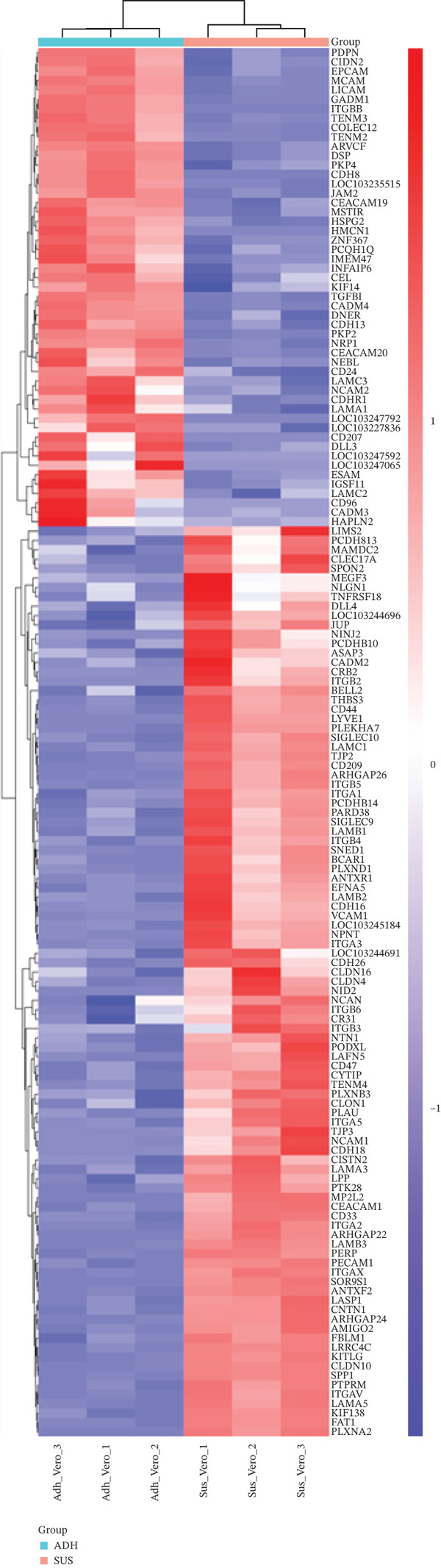
(d)

### 3.2. GO and KEGG Enrichment Analyses

To elucidate the biological functions of the screened DEGs, GO and KEGG pathway enrichment analyses were performed. The GO enrichment analysis identified 88 BPs, 33 cellular components, and 26 molecular functions associated with the DEGs. The top five significantly enriched BPs included cell adhesion, cell–cell adhesion, homophilic cell adhesion via plasma membrane adhesion molecules, cell–matrix adhesion, and the integrin‐mediated signaling pathway. The top five enriched cellular components were focal adhesion, plasma membrane, integrin complex, adherens junction, and cell surface. The top five enriched molecular functions comprised binding, calcium ion binding, cadherin binding, cell adhesion molecule binding, and cell–cell adhesion mediator activity (Figure 3a, Table 2). KEGG pathway analysis revealed that the DEGs were involved in extracellular matrix (ECM)–receptor interaction, cell adhesion molecules, focal adhesion, arrhythmogenic right ventricular cardiomyopathy, signaling pathways, human papillomavirus infection, cytoskeleton in muscle cells, small cell lung cancer, leukocyte transendothelial migration, hypertrophic cardiomyopathy, dilated cardiomyopathy, amoebiasis, and regulation of the cytoskeleton (Figure 3b, Table 3).

Figure 3GO and KEGG enrichment analyses of DEGs from Vero cells and *Chlorocebus sabaeus* adhesion genes. (a) Enriched GO terms of DEGs in DEGs from Vero cells and *C. sabaeus* adhesion genes; (b) KEGG pathway enrichment results for DEGs from Vero cells and *C. sabaeus* adhesion genes.

**Figure:**
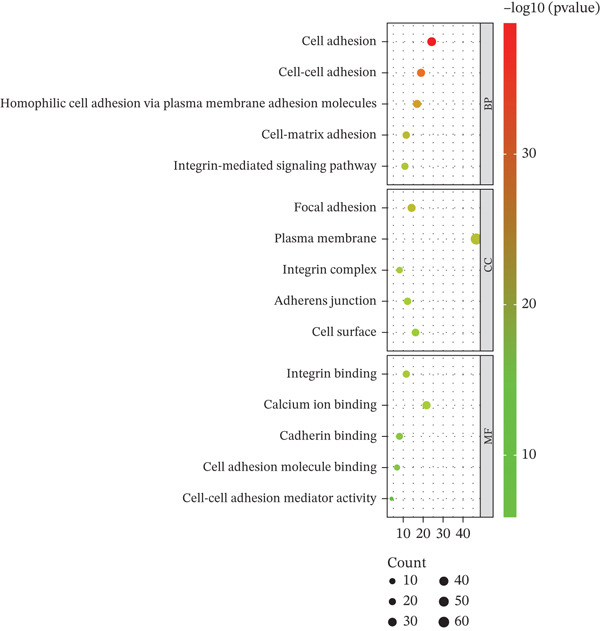
(a)

**Figure:**
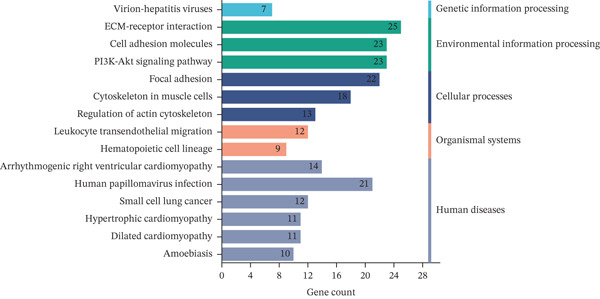
(b)

**Table 2:** GO enrichment analysis of the Top 10 terms.

Category	ID	Term	GeneRatio	*p*
BP	GO:0098609	Cell–cell adhesion	18.91892	3.07e − 32
	GO:0007156	Homophilic cell adhesion via plasma membrane adhesion molecules	16.89189	2.36e − 25
	GO:0007155	Cell adhesion	24.32432	2.69e − 39
	GO:0007160	Cell–matrix adhesion	11.48649	4.05e − 20
	GO:0007229	Integrin‐mediated signaling pathway	10.81081	3.83e − 18

CC	GO:0005925	Focal adhesion	14.18919	1.99e − 20
	GO:0005886	Plasma membrane	46.62162	1.83e − 19
	GO:0008305	Integrin complex	8.10811	4.38e − 17
	GO:0005912	Adherent junction	12.16216	5.43e − 17
	GO:0009986	Cell surface	16.21622	2.30e − 15

MF	GO:0005178	Integrin binding	11.48649	1.97e − 17
	GO:0005509	Calcium ion binding	21.62162	6.86e − 17
	GO:0045296	Cadherin binding	8.10811	6.00e − 12
	GO:0050839	Cell adhesion molecule binding	6.75676	2.16e − 11
	GO:0098632	Cell–cell adhesion mediator activity	4.05405	1.93e − 06

**Table 3:** KEGG enrichment analysis of the Top 15 terms.

Category	ID	Term	GeneRatio	*p*
KEGG	csab04512	ECM–receptor interaction	16.89189	3.25e − 28
	csab04514	Cell adhesion molecules	15.54054	1.97e − 19
	csab04510	Focal adhesion	14.86486	1.43e − 15
	csab05412	Arrhythmogenic right ventricular cardiomyopathy	9.45946	5.35e − 12
	csab04151	PI3K/Akt signaling pathway	15.54054	7.47e − 12
	csab05165	Human papillomavirus infection	14.18919	8.02e − 11
	csab04820	Cytoskeleton in muscle cells	12.16216	3.11e − 10
	csab05222	Small‐cell lung cancer	8.10811	6.55e − 09
	csab04670	Leukocyte transendothelial migration	8.10811	4.15e − 08
	csab05410	Hypertrophic cardiomyopathy	7.43243	9.28e − 08
	csab05414	Dilated cardiomyopathy	7.43243	2.94e − 07
	csab05146	Amoebiasis	6.75676	2.16e − 06
	csab04810	Regulation of the actin cytoskeleton	8.78378	4.67e − 06
	csab03272	Virion–hepatitis viruses	4.72973	9.06e − 06
	csab04640	Hematopoietic cell lineage	6.08108	1.39e − 05

### 3.3. Establishment and Analysis of PPI Networks

We used the STRING database and Cytoscape software to construct a PPI network with a comprehensive score threshold greater than 0.4, comprising 89 nodes and 738 edges (Figure 4a). The MCODE plug‐in in Cytoscape identified four closely connected gene modules, including 38 nodes and 386 interaction pairs (Figures 4b, 4c, 4d, and 4e). GO enrichment analysis revealed that these genes were primarily involved in cell adhesion, cell–cell adhesion, cell–matrix adhesion, the integrin‐mediated signaling pathway, the integrin complex, focal adhesion, cell surface components, integrin binding, cell–cell adhesion mediator activity, and collagen binding related to cell–matrix adhesion (Figure 4f). KEGG pathway analysis indicated that these genes were mainly associated with ECM–receptor interaction, focal adhesion, cell adhesion molecules, human papillomavirus infection, and related pathways (Figure 4g).

Figure 4(a) PPI network constructed using the STRING database. (b–e) Four important gene clustering modules. (f) GO analysis of the modular genes. (g) KEGG enrichment analysis of the modular genes.

**Figure:**
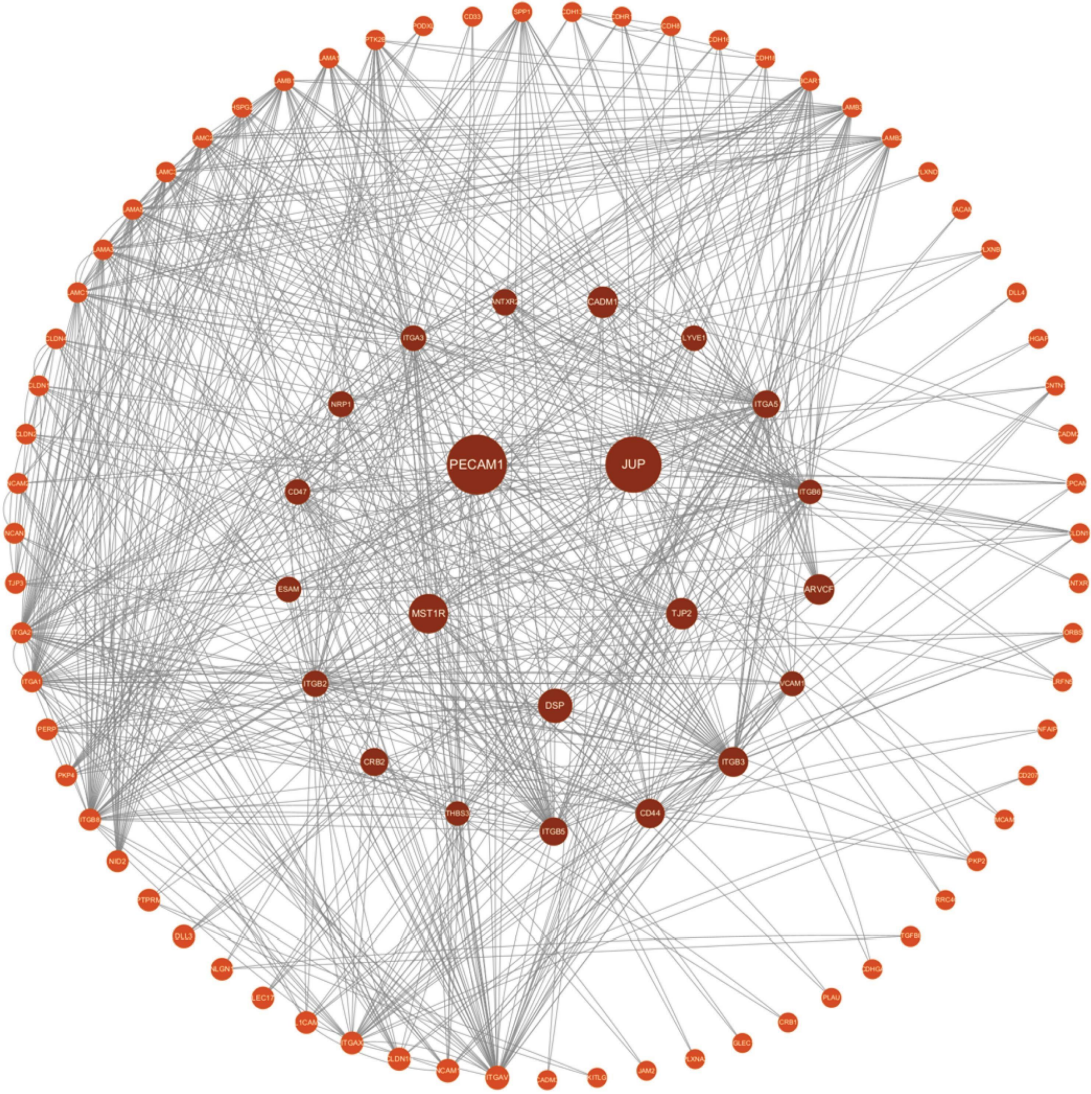
(a)

**Figure:**
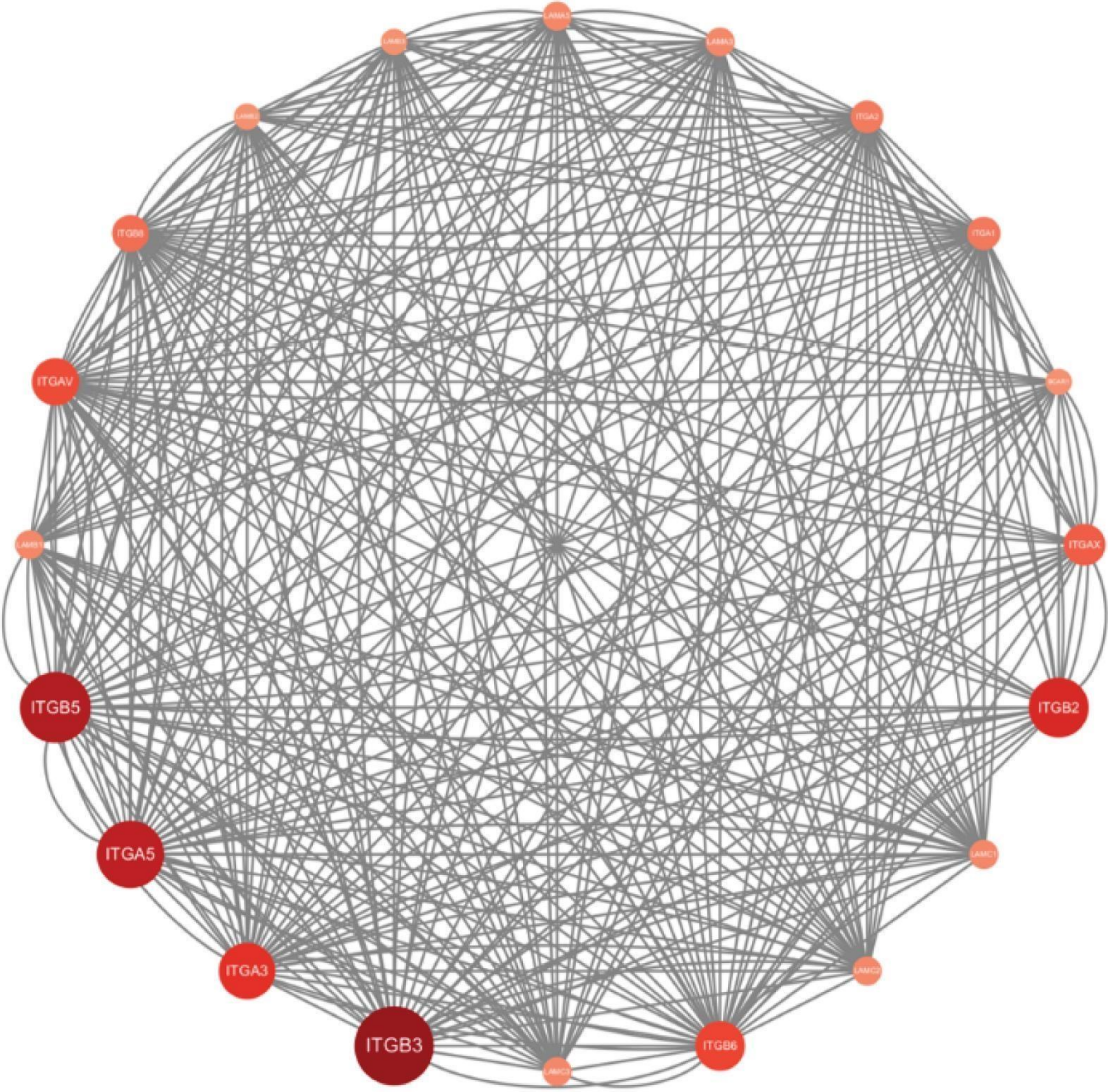
(b)

**Figure:**
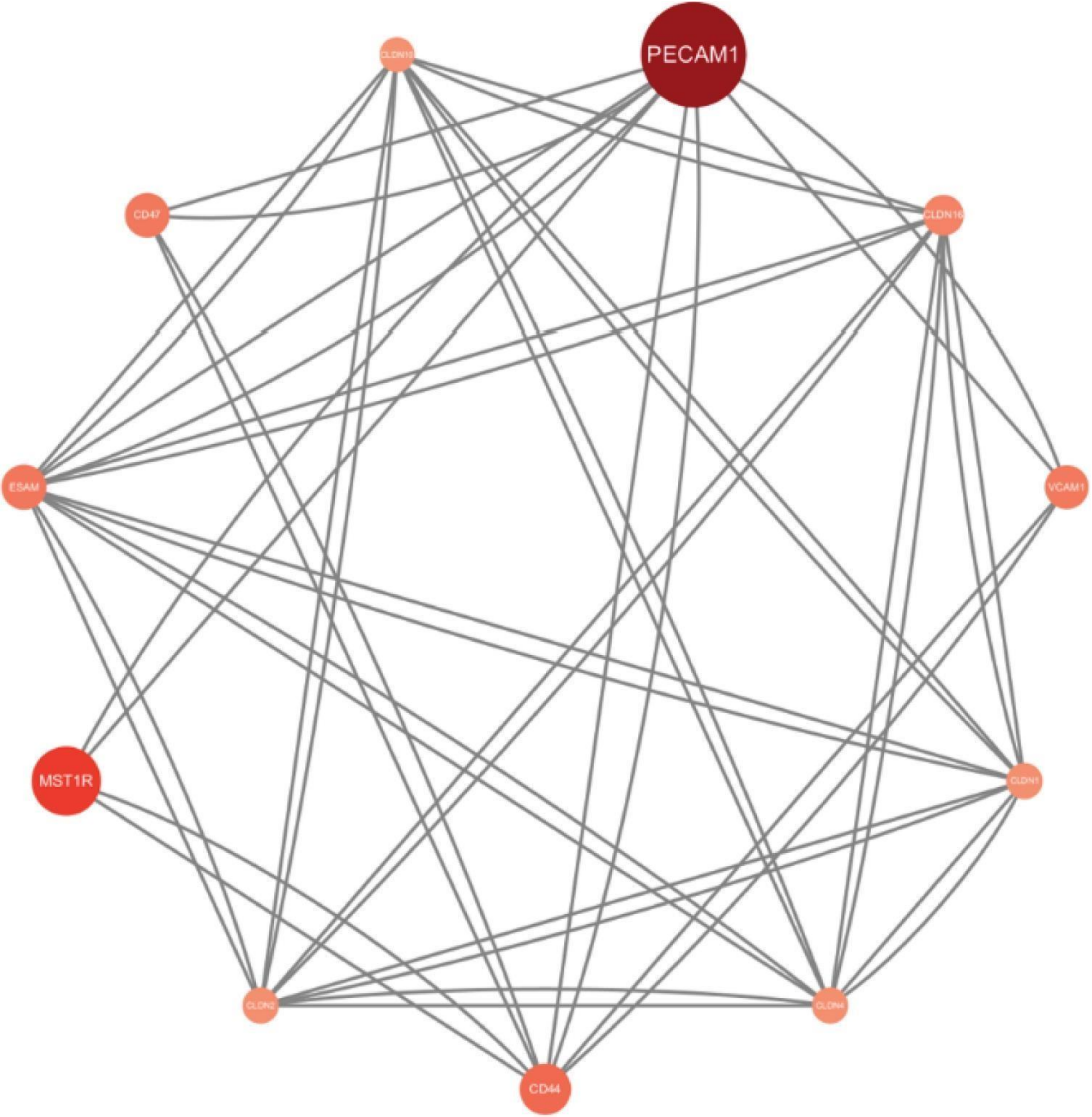
(c)

**Figure:**
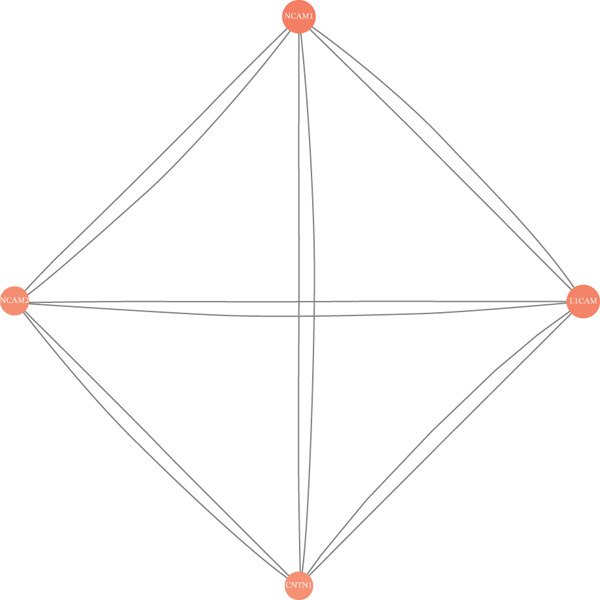
(d)

**Figure:**
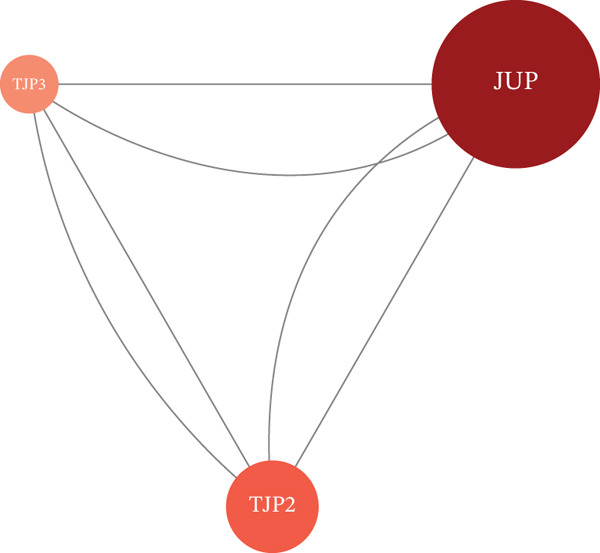
(e)

**Figure:**
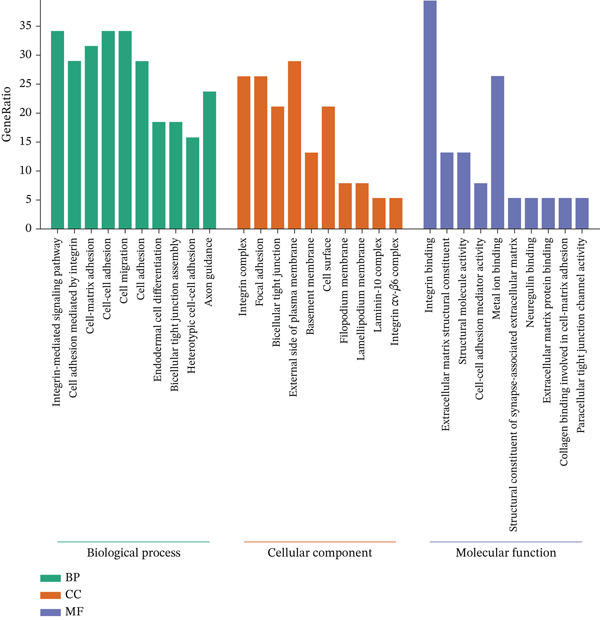
(f)

**Figure:**
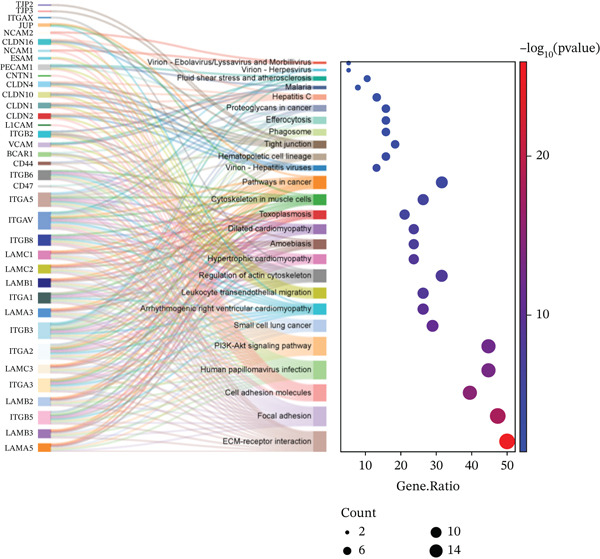
(g)

### 3.4. Selection and Analysis of Core DEGs

Using six algorithms from the CytoHubba plug‐in, the Top 20 hub genes were identified (Table 4). Venn diagram analysis revealed six overlapping hub genes: *ITGA3*, *ITGA5*, *ITGB3*, *ITGB5*, junctional plakoglobin (*JUP*), and platelet endothelial cell adhesion molecule‐1 (*PECAM1*) (Figure 5a). GSEA indicated that these genes are primarily associated with intercellular adhesion, cell migration, and the cell cycle (Figures 5b, 5c, 5d, 5e, 5f, and 5g). The log2 fold change (log2FC) values and the significance of the expression levels for each group of six hub genes are presented in Table 5.

**Table 4:** Top 20 hub genes identified by six CytoHubba algorithms.

Degree	Closeness	Stress	Radiality	Betweenness	Eccentricity
ITGB3	ITGB3	JUP	PECAM1	PECAM1	PECAM1
ITGB5	ITGB5	DSP	ITGB3	JUP	JUP
ITGA5	ITGA5	ARVCF	ITGB5	MST1R	DSP
ITGAV	ITGAV	PECAM1	ITGA5	DSP	PKP4
ITGB6	ITGB6	NID2	ITGB2	TJP2	PERP
ITGB8	PECAM1	CRB2	ITGAV	CADM1	PKP2
ITGA3	ITGB2	ITGB3	CD44	ARVCF	ITGB3
ITGA1	ITGA3	ITGB5	ITGB6	ITGB3	ITGB5
ITGA2	ITGB8	TJP2	ITGA3	CD44	ITGA5
ITGB2	ITGA2	DLL3	MST1R	ITGB5	ITGAV
LAMA5	ITGA1	PERP	VCAM1	CRB2	ITGB6
LAMA3	CD44	ANTXR2	JUP	ITGA5	ITGA3
LAMC1	MST1R	ITGB6	CD47	ITGB2	ITGB2
LAMB1	CD47	ITGA3	DSP	NRP1	CD44
CD44	JUP	ITGA5	ITGB8	ESAM	MST1R
LAMC2	VCAM1	ITGAV	ITGA2	ITGA3	CD47
LAMC3	LAMA5	LAMC1	ITGA1	LYVE1	VCAM1
PECAM1	LAMA3	LAMA5	THBS3	CD47	ARVCF
LAMB3	ITGAX	LAMA3	ITGAX	ANTXR2	ESAM
JUP	DSP	LAMC2	ESAM	VCAM1	CLDN1

Figure 5Screening core genes and functional pathway enrichment. (a) Venn diagram showing six overlapping cell adhesion–related genes identified by six different algorithms. (b) GSEA functional analysis of core cell adhesion–related genes in Vero cells, highlighting JUP. (c) PECAM1, (d) ITGA3, (e) ITGA5, (f) ITGB3, (g) ITGB5.

**Figure:**
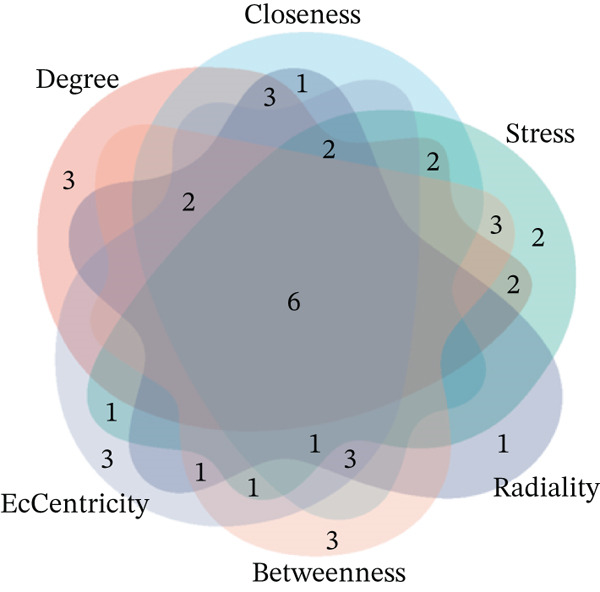
(a)

**Figure:**
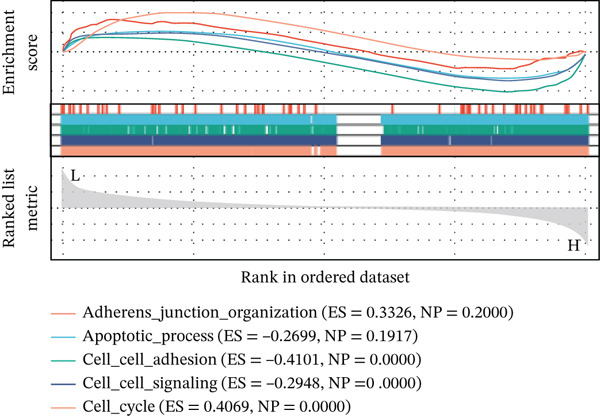
(b)

**Figure:**
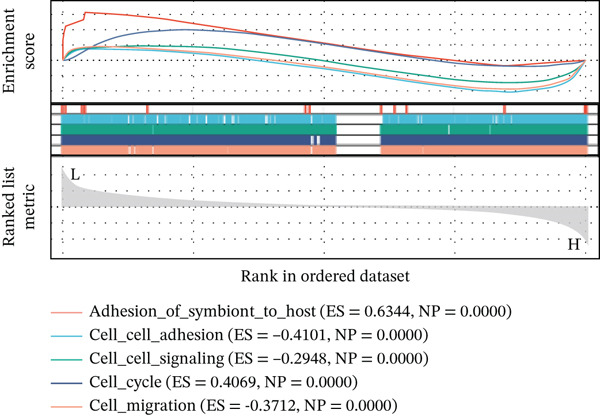
(c)

**Figure:**
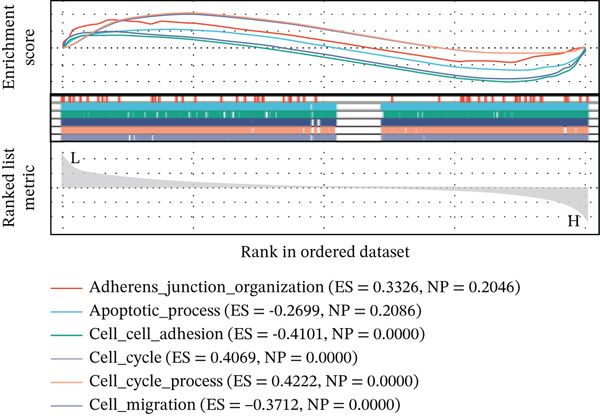
(d)

**Figure:**
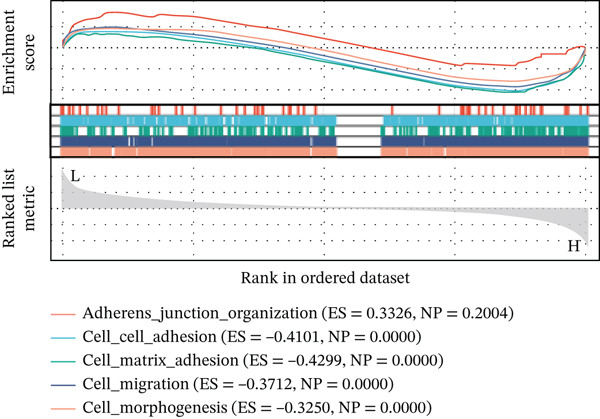
(e)

**Figure:**
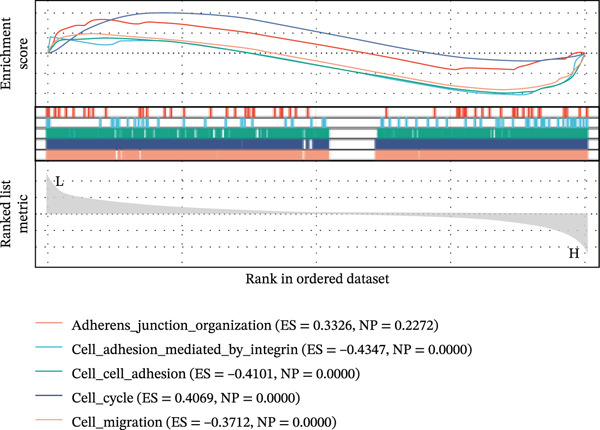
(f)

**Figure:**
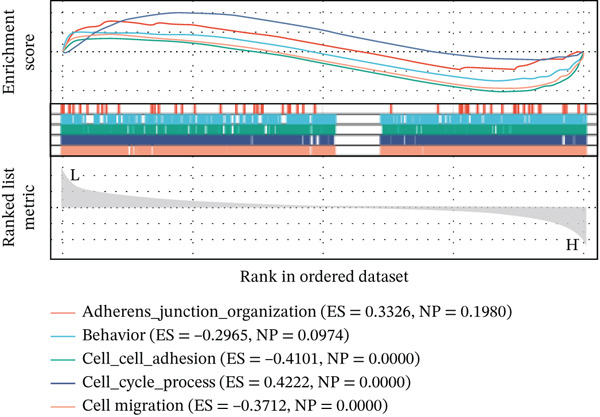
(g)

**Table 5:** Six hub genes identified by Venn diagram analysis using six algorithms of the CytoHubba plug‐in.

Gene ID	Adhesion expression			Suspension expression			*p* values	log2 fold change
	1	2	3	1	2	3		
JUP	0.758486	1.41165	0.864439	2.23293	2.08216	2.07271	8.40e − 08	−1.137753
PECAM1	0	0.021041	0.039912	0.607531	0.628668	0.640536	3.86e − 26	5.013517
ITGA3	132.843	119.546	118.318	829.719	587.535	591.939	2.75e − 10	−2.56803
ITGA5	27.3024	21.4973	24.4302	70.0812	98.4718	97.3888	4.41e − 24	−1.984127
ITGB3	104.158	90.2275	101.999	128.496	221.759	195.278	0.000136	1.028469
ITGB5	12.6381	13.523	11.8153	52.3827	44.3499	46.0618	1.62e − 23	2.045102

### 3.5. qRT‐PCR Analysis

**Figure 6:**
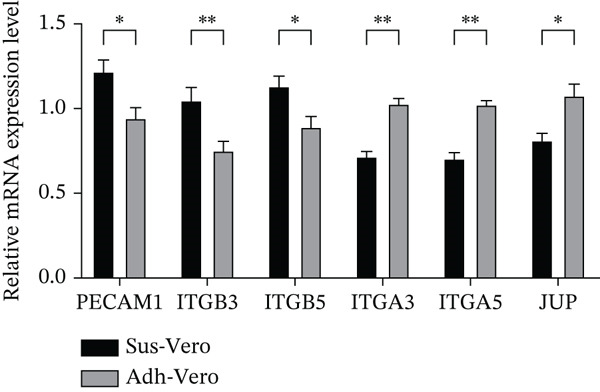
qRT‐PCR validation of the expression levels of six core hub genes *n* = 3;  ^∗^ *p* < 0.05,  ^∗∗^ *p* < 0.01,  ^∗∗∗^ *p* < 0.001; and ns, *p* > 0.05.

To verify the accuracy of the transcriptome sequencing data, qRT‐PCR was performed on six selected genes, using *GAPDH* as the internal reference gene for normalization. The results showed that the expression patterns of *PECAM1*, *JUP*, *ITGA3*, *ITGA5*, *ITGB5*, and *ITGB3* were consistent with the omics data (Figure 6).

## 4. Discussion

Cell adhesion is fundamental for multicellular organisms to maintain tissue structure, facilitate intercellular communication, and regulate physiological functions [24]. The expression levels of cell adhesion–related genes can influence these functional characteristics [25]. In this study, we systematically analyzed the gene expression profiles of adherent and suspended Vero cells and investigated their transcriptomic features using bioinformatics tools. Ultimately, six potential target genes and key pathways were identified (Figure 5). During the functional enrichment analysis of DEGs, we identified three pathways related to cell adhesion: ECM–receptor interaction, cell adhesion molecules, and focal adhesion. ECM–receptor interaction refers to the binding between the ECM and cell surface receptors, which plays a crucial role in regulating cell behavior and phenotype [26]. Previous studies have demonstrated that ECM adhesion to cells effectively forms a bridge between the ECM and the actin cytoskeleton by assembling a supramolecular protein complex, which is vital for bidirectional signal transduction between extracellular molecules and the cytoplasm [27]. Cell adhesion molecule–mediated signaling pathways are key regulators in various physiological and pathological processes [28]. Based on their structural characteristics, these molecules are primarily classified into four protein families: integrins, selectins, the immunoglobulin superfamily, and cadherins [29]. Integrins bind to ligands to activate the Ras‐MAPK and PI3K/Akt signaling pathways via FAK and Src kinases, thereby regulating cell proliferation and adhesion [30]. Focal adhesions, as central hubs of cell–ECM interactions, have recently yielded significant insights into the molecular mechanisms of cell adhesion. Overexpression of FAK can promote epithelial–mesenchymal transition (EMT), enhancing ECM remodeling and cellular invasion [31].

*JUP* is a crucial member of the catenin family [32]. It binds tightly and mutually exclusively to the intracellular domain of cadherin and connects to the actin cytoskeleton via *α*‐catenin, thereby maintaining cell‐to‐cell adhesion [33]. Due to *JUP*′s essential role in adhesion and tight junctions, cells with low *JUP* expression exhibit decreased adhesion stability, detach easily from culture substrates, and have a low recovery rate after passage. However, these cells display improved single‐cell dispersion and enhanced metabolic activity, making them suitable for suspension adaptation and short‐term rapid expansion. Conversely, they become more sensitive to shear forces and nutrient fluctuations in the culture environment, and their phenotype is prone to EMT [34, 35]. Overexpression of *JUP* significantly enhances the structural stability of intercellular desmosomes and adherens junctions, strengthens intercellular adhesion and resistance to shear stress, and preserves the phenotypic integrity of epithelial cells. Additionally, it reduces the nuclear translocation of *β*‐catenin by competitively binding to E‐cadherin, thereby inhibiting cell migration and abnormal proliferation and effectively lowering the risk of EMT. At the process level, cells with high *JUP* expression adhere firmly to culture surfaces, resulting in excellent monolayer stability. Their survival in serum‐free, chemically defined, nutrient‐limited media is more stable, making them suitable for long‐term adherent culture, 3D tissue construction, and large‐scale bioproduction. Tissue engineering scaffolds constructed from these cells exhibit higher mechanical strength and improved barrier function. The Vero cell line, commonly used for viral vaccine production, has also demonstrated enhanced process stability and culture cycle tolerance with increased *JUP* expression, although cell proliferation rates have slowed. Excessively high *JUP* expression can lead to dense cell aggregation, which reduces mass transfer and oxygen supply efficiency within the culture system [36, 37]. Currently, there are few reports on the suspension adaptation of cell lines with *JUP* overexpression, knockdown, or knockout, although such approaches are theoretically feasible.

*PECAM1* is a crucial member of the immunoglobulin superfamily of adhesion molecules. It is widely expressed in endothelial cells, leukocytes, and other cell types and serves as a specific marker of vascular endothelial cells [38]. *PECAM1* mediates cell adhesion and enhances integrin function [39]. The core conclusion of the PECAM1‐Pyk2 axis regulating the anchorage‐independent growth of tumor cells, as proposed by Zhang et al., is highly consistent and further extends the mechanism to dynamic suspension culture systems. It is evident that homophilic adhesion of *PECAM1* is a prerequisite for its intracellular phosphorylation and signal activation. Cell suspension aggregation was completely inhibited following *PECAM1* knockdown, directly indicating that PECAM1‐mediated intercellular adhesion is the initial step in suspension adaptation. Unlike integrin‐dependent matrix adhesion signals, *PECAM1* establishes an independent survival signal through intercellular homophilic interactions, bypassing ECM dependence and enabling cells to resist anoikis. This represents a unique molecular mechanism by which *PECAM1* regulates suspension adaptation [40]. As a classic mechanoreceptor in vascular endothelial cells, *PECAM1* senses fluid shear stress in suspension culture, activates the ERK/PI3K/Akt prosurvival pathway, enhances cellular mechanical stress tolerance, and integrates antiapoptotic and mechanical adaptation functions. This makes *PECAM1* a key hub molecule for cellular adaptation to complex suspension microenvironments [41]. *PECAM1* is a critical positive regulator of cell suspension adaptation. By mediating cell aggregation, inhibiting anoikis, and sensing mechanical stress, *PECAM1* facilitates anchorage‐independent growth and successful suspension adaptation. The PECAM1‐Pyk2 signaling axis is a promising target for fundamental research on anchorage‐independent growth, optimization of industrial cell culture, and antitumor metastasis therapies.

Integrins are heterodimeric cell surface receptors that mediate mechanical linkage between cells and the ECM [42]. The core subunits of the integrin family—*ITGB3*, *ITGB5*, *ITGA3*, and *ITGA5*—facilitate adhesion and signal transduction between cells and the ECM by forming heterodimers [43]. *ITGA5* typically pairs with *ITGB1* to form a dimer that functions as a classic cell–fibronectin adhesion receptor, transmitting survival signals via the FAK‐PI3K/Akt pathway. It plays a crucial role in enabling adherent cells to resist anoikis. Mostafavi‐Pour et al. used hanging drop culture to aggregate human Wharton′s jelly stem cells (hWJSCs) into 3D spheres and observed a significant decrease in *ITGA5* gene expression [44], indicating that *ITGA5* acts as a negative regulator of suspension adaptation. *ITGA3* primarily binds to laminin and collagen, maintaining the epithelial phenotype by regulating the FAK‐MAPK signaling pathway. *ITGB3* enhances antiapoptotic capacity mainly through the FAK‐Akt/ERK signaling pathway. *ITGB5* predominantly binds to fibronectin and modulates FAK‐Rac1 signaling, thereby influencing cell migration and survival. Although these genes are rarely targeted for cell suspension adaptation, they represent promising candidates. Moreover, inducing suspension growth in adherent cells may be achieved by simultaneously regulating the expression levels of these four genes.

## 5. Conclusion

In summary, we analyzed the transcriptomes of adherent and suspension Vero cells using bioinformatics methods and identified six hub genes (*ITGA3*, *ITGA5*, *ITGB3*, *ITGB5*, *JUP*, and *PECAM1*) along with several pathways related to cell adhesion. This study highlights key functional candidate targets for genetic engineering aimed at adapting suspension Vero cells from adherent Vero cell lines.

## Author Contributions

J.B., the corresponding author of this article, designed the project and made suggestions for some of the contents of this article. Y.F. designed and conducted experiments and completed the manuscript writing with the support of D.Z., Q.L., Y.W., R.B., Y.Z., and W.G., who helped analyze and discuss the results.

## Funding

This study was supported by the Northwest University for Nationalities Central University Fundamental Research Business Funding Project, grant number 31920190003; the National Natural Science Foundation of China, grant number 31860696; the Lanzhou Talent Entrepreneurship and Innovation Project, grant number 2020‐RC‐85; and the Special Fund Project for Basic Scientific Research Funds for the Central Universities, grant numbers 31920230154 and 31920250002.

## Disclosure

All authors review and approve the final manuscript.

## Ethics Statement

The authors have nothing to report.

## Consent

The authors have nothing to report.

## Conflicts of Interest

The authors declare no conflicts of interest.

## Data Availability

Data are available upon request due to privacy/ethical restrictions.
